# Parallel Evolution of Polydactyly Traits in Chinese and European Chickens

**DOI:** 10.1371/journal.pone.0149010

**Published:** 2016-02-09

**Authors:** Zebin Zhang, Changsheng Nie, Yaxiong Jia, Runshen Jiang, Haijian Xia, Xueze Lv, Yu Chen, Junying Li, Xianyao Li, Zhonghua Ning, Guiyun Xu, Jilan Chen, Ning Yang, Lujiang Qu

**Affiliations:** 1 Department of Animal Genetics and Breeding, National Engineering Laboratory for Animal Breeding, College of Animal Science and Technology, China Agricultural University, Beijing, 100193, China; 2 Beijing Municipal General Station of Animal Science, Beijing, 100107, China; 3 College of Animal Science and Technology, Anhui Agricultural University, Hefei, 230036, China; 4 College of Animal Science, Shandong Agricultural University, Taian, 271018, China; 5 Institute of Animal Science, Chinese Academy of Agricultural Sciences, Beijing, 100193, China; China Agricultural Univeristy, CHINA

## Abstract

Polydactyly is one of the most common hereditary congenital limb malformations in chickens and other vertebrates. The zone of polarizing activity regulatory sequence (ZRS) is critical for the development of polydactyly. The causative mutation of polydactyly in the Silkie chicken has been mapped to the ZRS; however, the causative mutations of other chicken breeds are yet to be established. To understand whether the same mutation decides the polydactyly phenotype in other chicken breeds, we detected the single-nucleotide polymorphism in 26 different chicken breeds, specifically, 24 Chinese indigenous breeds and 2 European breeds. The mutation was found to have fully penetrated chickens with polydactyly in China, indicating that it is causative for polydactyly in Chinese indigenous chickens. In comparison, the mutation showed no association with polydactyly in Houdan chickens, which originate from France, Europe. Based on the different morphology of polydactyly in Chinese and European breeds, we assumed that the trait might be attributable to different genetic foundations. Therefore, we subsequently performed genome-wide association analysis (GWAS) to locate the region associated with polydactyly. As a result, a ~0.39 Mb genomic region on GGA2p was identified. The region contains six candidate genes, with the causative mutation found in Chinese indigenous breeds also being located in this region. Our results demonstrate that polydactyly in chickens from China and Europe is caused by two independent mutation events that are closely located in the chicken genome.

## Introduction

The chicken (*Gallus gallus*) is a modern descendant of the dinosaurs and the first non-mammalian amniote [1]. This species is one of the most common and widespread agricultural animals, providing essential proteins for the human food industry. In addition, it is the main laboratory model for human disease and genetics analysis [2, 3].

Polydactyly is one of the most common hereditary congenital limb malformations in humans, chickens, mice, and other vertebrates that have supernumerary fingers and/or toes [4–7]. Although the number of extra fingers/toes is subject to variability, polydactyly is classified as a rare qualitative trait [8]. High rates of polydactyly are attributed to its autosomal-dominant trait [9]. For instance, polydactyly occurs in 5 to 19 cases of every 10,000 live births in humans [10]. This high incidence in humans has resulted in focused research, with the chick limb becoming a classical model for studying developmental mechanisms and pattern formation during the embryogenesis of vertebrate embryos, due to its size and accessibility [11].

In general, chickens have four toes on each foot, but some breeds have more, such as the Beijing-You, Silkie, Jiningbairi, Dorking, and Houdan. The additional toes in polydactylous breeds of fowl do not represent the restoration of the fifth digit lost from the typical pentadactyl foot of higher vertebrates; rather, it is the result of the development of new toes on the opposite side of the foot [9]. Classic embryonic studies of the chick limb have revealed that the growth and patterning along the proximal/distal (P/D), anterior/posterior (A/P), and dorsal/ventral (D/V) axes are controlled by 3 signaling centers, specifically, the apical ectodermal ridge (AER), the zone of polarizing activity (ZPA), and the limb bud ectoderm [12]. For instance, chick limb A/P patterning is controlled by the secretion of the sonic hedgehog (*SHH*) protein by a small group of cells in the ZPA, which is located on the posterior mesoderm [13, 14]. The overexpression of *SHH* throughout the mesoderm of developing chicken limbs causes mutant polydactyly [15]. In comparison, low *SHH* expression, to the extent that it is not detectable in developing limbs, causes mutant oligozeugodactyly [13]. A *cis*-regulator of *SHH* has been identified in human preaxial polydactyly (PPD), and is responsible for the initiation and spatially specific expression of *SHH* in the ZPA. This regulator is located in intron 5 of the *LMBR1* gene, which is located 1 Mb upstream of *SHH* [16]. The intron 5 sequence of *LMBR1*, which represents the ZPA regulatory sequence (ZRS), is conserved in humans, mice, pufferfish (fugu), and chickens [17–20]. A few point mutations in the ZRS region have been identified as being responsible for human limb malformation, including the presence of a triphalangeal thumb and PPD [21–25].

Various genetic mechanisms underlie chicken polydactyly phenotypes. In the Silkie chicken, SNPs in the ZRS region of *LMBR1* intron 5 lead to ectopic *SHH* expression during embryonic development, resulting in polydactyly and oligozeugodactyly [15, 26, 27]. In the Dorking chicken, polydactyly is caused by the up-regulation of *FGF4* expression, rather than that of *SHH* [28]. Through linkage analysis and GWAS, polydactyly has been mapped to genomic regions on GGA2, with several candidate genes potentially responsible for this trait in the Beijing-You chicken [29–31]. However, the genetic background for polydactyly has yet to be elucidated in other chicken breeds.

In this study, we discriminated the polydactyly phenotypes of 26 chicken breeds and identified the genetic basis for the traits. Our results are expected to provide new insights about the evolution of polydactyly in chicken breeds, which is expected to contribute towards human research on this topic.

## Materials and Methods

### Ethics statement

The entire procedure was carried out in strict accordance with the protocol approved by the Animal Welfare Committee of China Agricultural University (Permit Number: XK622).

### Sampling and DNA extraction

We selected four polydactylous chicken breeds, including three Chinese indigenous breeds (Beijing-You, Silkie, and Jiningbairi) and one European breed (Houdan) for which the polydactyly trait has not yet been elucidated. We also selected 21 local Chinese breeds with high genetic diversity of each other from 21 provinces [32] and White Leghorn as non-polydactyly comparisons. The sampling datasets are summarized in Table 1. Blood samples were collected from the brachial veins of chickens by standard venipuncture. Genomic DNA was extracted from blood using the standard phenol/chloroform method. DNA quality was controlled using the NanoDrop 2000 spectrophotometer (Thermo Fisher Scientific, USA).

**Table 1:** Genotypes of the causative mutation and their distribution in 26 chicken breeds.

	Number							
Breed Name	Total	Polydactyly			Non-polydactyly			Origin
		CC	AC	AA	CC	AC	AA	
Beijing-You	236	0	51	4	181	0	0	China
Silkie	110	0	39	62	9	0	0	China
Jiningbairi	38	0	18	0	20	0	0	China
Houdan	86	43	0	0	43	0	0	France
White Leghorn	96	0	0	0	96	0	0	Italy
Langya	10	0	1	0	9	0	0	China
Hetian	10	0	0	0	10	0	0	China
Rugao	10	0	0	0	10	0	0	China
Mahuang	10	0	0	0	10	0	0	China
Xianju	10	0	0	0	10	0	0	China
Gushi	10	0	0	0	10	0	0	China
Turpan Game	10	0	0	0	10	0	0	China
Hongshan	10	0	0	0	10	0	0	China
Shanbei	10	0	0	0	10	0	0	China
Nandan Yao	10	0	0	0	10	0	0	China
Jiuyuan Black	10	0	0	0	10	0	0	China
Tibetan	10	0	0	0	10	0	0	China
Haidong	10	0	0	0	10	0	0	China
Yangshan	10	0	0	0	10	0	0	China
Jingning	10	0	0	0	10	0	0	China
Yanjin Black	10	0	0	0	10	0	0	China
Wenchang	10	0	0	0	10	0	0	China
Hebei Domestic	10	0	0	0	10	0	0	China
Sanhui	10	0	0	0	10	0	0	China
Huanglang	10	0	0	0	10	0	0	China
Pudong	10	0	0	0	10	0	0	China

### Polydactyly phenotype identification

**Fig 1:**
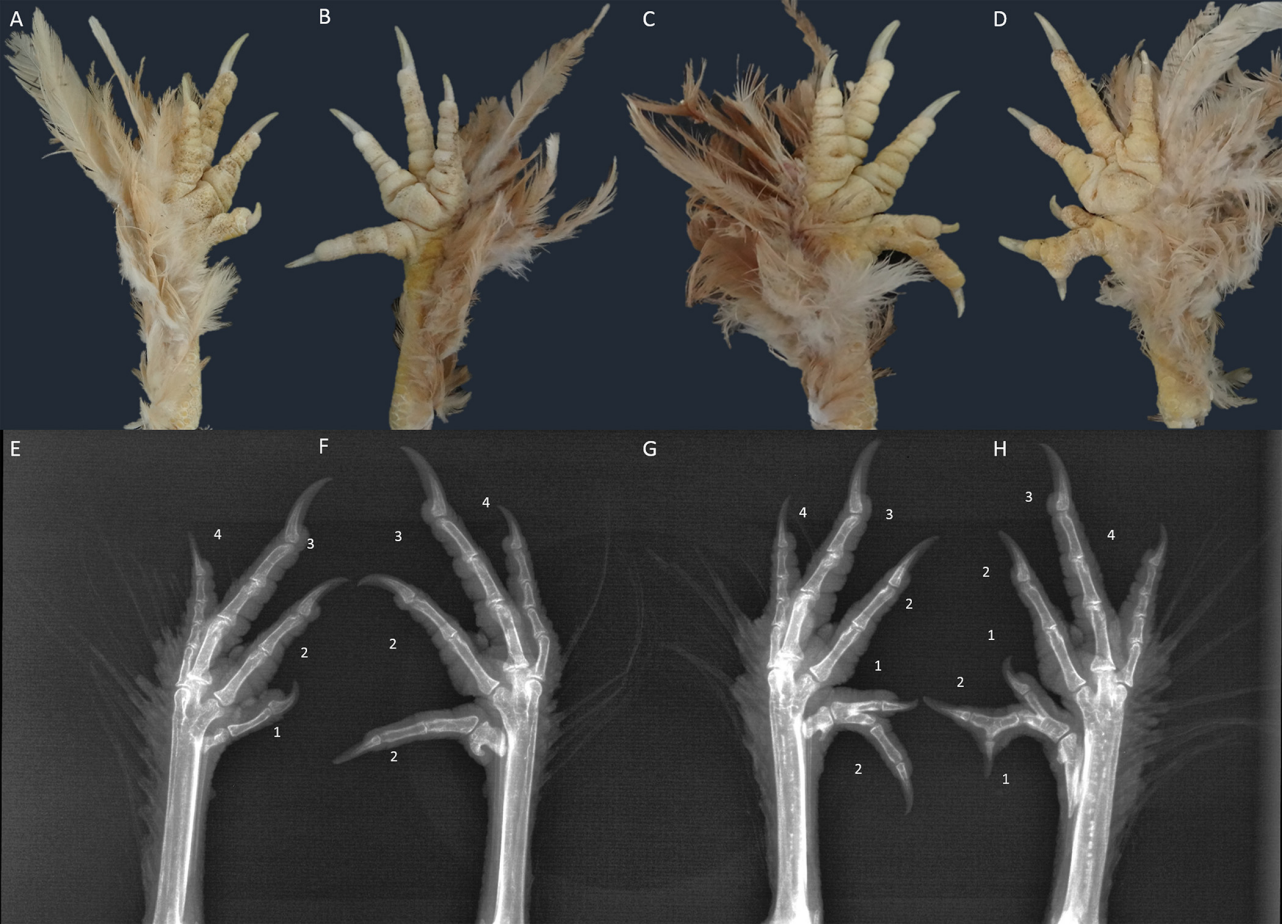
Variation in X-ray digital radiography with respect to Beijing-You foot polydactyly. **A-D** show the different foot phenotypes of Beijing-You chicken, **E-H** show the X-ray digital radiography coordinately with **A–D**. **A & E:** non-polydactylous foot with four digits, identified from anterior to posterior (1, 2, 3, 4). **B & F**: Beijing-You foot with four digits, with a polyphalange in digit “1” making it digit “2” (2, 2, 3, 4). **C & G**: polydactylous Beijing-You foot with five digits (2, 1, 2, 3, 4). **D & H**: polydactylous Beijing-You foot with six digits (1, 2, 1, 2, 3, 4).

For non-polydactyly birds with four digits on each foot, the digits were labeled 1 to 4 from the anterior to posterior position on the foot (Fig 1). Chickens with more than four toes on one or both feet were designated as having the polydactyly phenotype by visual inspection and X-ray digital radiography. Some birds had four toes, but the anterior-most toe had an extra phalanx that was termed the polyphalange, and was considered a variation of polydactyly in the analyses of the present study [9].

### Linkage analysis of causative SNP and diagnostic PCR

An SNP (ss161109890) identified in the intronic ZRS region of *LMBR1*, which is the *cis*-regulatory element of *SHH*, has been confirmed to cause Silkie polydactyly [26]. We first analyzed the SNP that has been found in all sampled Silkie chickens by using a diagnostic PCR method. We developed a diagnostic PCR-restriction fragment length polymorphism (PCR-RFLP) method with forward (5ʹ-GCGATTTCCTCTCACCCACA-3ʹ) and reverse (5ʹ-AGCTGAGCAACATGACAGCA-3ʹ) primers, producing a 389-bp fragment. When the genotype is CC, the PCR product is digested into two fragments (245 bp and 144 bp) by the restriction enzyme *BsrDI*. Some of the PCR products were also sequenced for verification. Furthermore, we analyzed the correlation between this SNP and polydactyly in all chicken breeds.

### GWAS for polydactyly in Houdans

All Houdan chickens were wild homozygous types at the causative mutation point, regardless of whether birds had polydactyly or not. To refine the genomic regions responsible for polydactyly in Houdan chickens, we performed genome-wide association studies using the 600 K Affymetrix Axiom Chicken Genotyping Array (Affymetrix, Inc. Santa Clara, CA, USA) by the case-control method, with 43 polydactylous and 43 non-polydactylous Houdan chickens.

### Genotyping and quality control

From an initial set of 580,961 SNPs [33], 8,374 SNPs with unknown genomic positions and 56 SNPs with redundant genomic coordinates were disregarded. Subsequently, first-pass quality control and genotype calling from the raw data in the form of CEL files were implemented with Affymetrix Power Tools v1.16.0 (APT) software using the Axiom GT1 algorithm. Only samples with a dish quality control (DQC) of 0.82 or more and a call rate of 98% were included in downstream analyses. An R script supplied by Affymetrix was run to compute SNP QC metrics and filter out individual SNPs falling below the given threshold. All parameters were set to the default values recommended by Affymetrix. After these QC steps, 86 samples and 546,124 SNPs remained. In addition, we excluded 26,656 SNPs on sex chromosomes because current statistical methods are more powerful at detecting the associations between phenotypes and autosomal genotypes. To improve the power of the association analyses, we disregarded a further 171,426 SNPs with minor allele frequency (MAF) < 1% and 441 SNPs that deviated from the Hardy-Weinberg equilibrium (HWE) test P < 10^−6^ using the PLINK v1.90 package [34]. Finally, a total of 86 samples and 369,020 SNPs were eligible for inclusion in the following GWAS.

### Association analysis

Prior to GWAS, we conducted a principal component analysis (PCA) to eliminate spurious associations due to potential cryptic relatedness or hidden population stratification. Considering that clusters of highly correlated SNPs may distort resulting PCs, we first pruned the full SNP set to 24,449 independent SNPs via the—indep-pairwise 25 5 0.2 command (PLINK), and then calculated the top 5 PCs as covariates in the mixed model. To establish proper thresholds for genome-wide suggestive and significant associations, we corrected for multiple testing using the simpleM method [35], which accounts for linkage disequilibrium (LD) relationships among SNPs. Using simpleM, we estimated the effective number of independent tests as Meff = 57,657; thus, the genome-wide suggestive and significant P-values were 1.73 × 10^−5^ (1.00/57,657) and 8.67 × 10^−7^ (0.05/57,657), respectively.

We performed univariate tests of association for SNPs that had MAF ≥ 0.05 using an exact mixed model approach implemented in the GEMMA v0.94 software [36]. The centered relatedness matrix was calculated based on these independent SNPs for all cases. Subsequently, each SNP was tested for additive association with each trait by modeling the effects of genotypes and the additional covariates, including the top 5 PCs as fixed effects and genetic relatedness as random effects, because our samples were from highly structured populations with strong family relatedness. The Manhattan plot depicting -log10-transformed observed P-values was generated using the “gap” package in R.

## Results

### Polydactyly phenotype differs between Chinese and European chicken breeds

**Fig 2:**
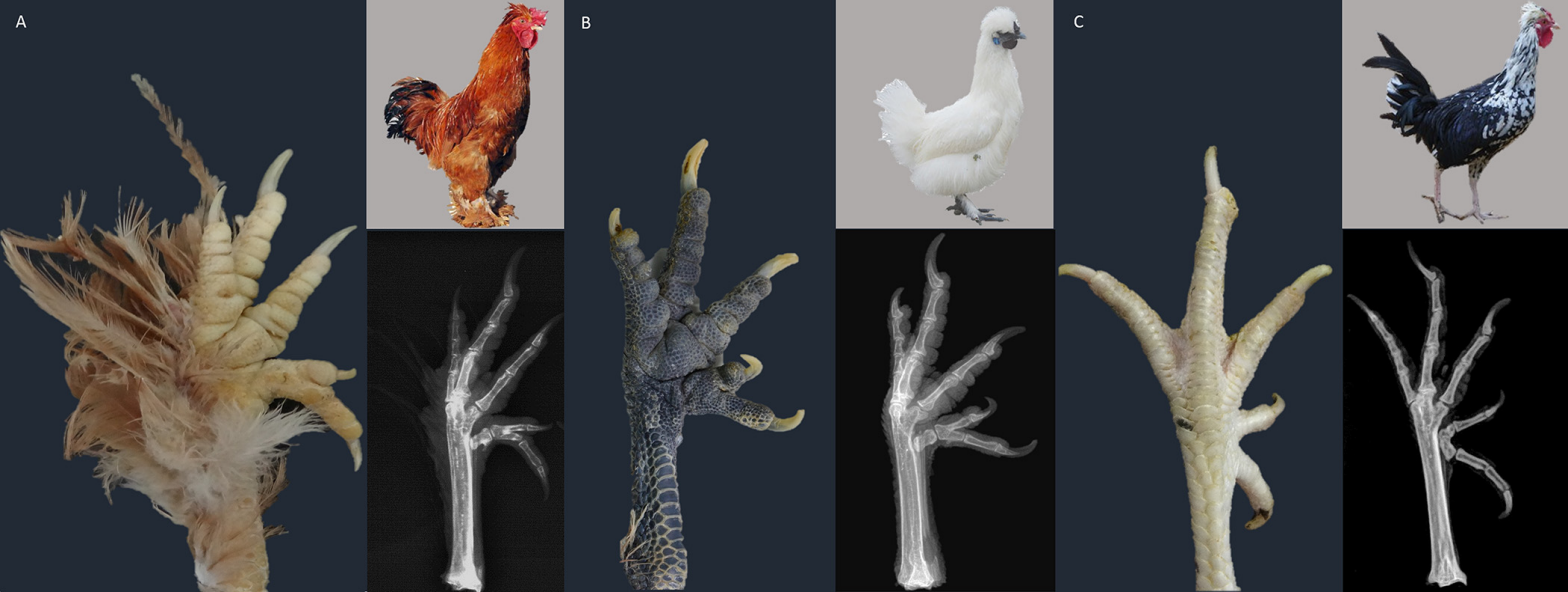
Principal polydactyly subtypes in Beijing-You, Silkie, and Houdan chickens. Two subtypes of polydactyly were found in the three chicken breeds, but with difference prevalence. Subtype I: principal subtype of Beijing-You (A) and Silkie (B), in which an extra toe was separated from the second phalanx in the most anterior toe; Subtype II: principal subtype of Houdan (C), with extra toe separation occurring from the first phalanx.

Polydactyly, including polyphalange, was detected in 55 out of 236 Beijing-You chickens, 101 out of 110 Silkie chickens, 18 out of 38 Jiningbairi chickens, and 43 out of 86 Houdan chickens (Table 1). The rest of the birds had the non-polydactyly phenotype (Table 1). In the four analyzed chicken breeds, we discovered two main subtypes of polydactyly (Fig 2). Subtype I is the presence of extra toes arising from the second phalanx of the most anterior toe, while subtype II is when extra toe separation originates from the first phalanx. Both subtypes were found in all three breeds, but with different prevalence. For Beijing-You, Silkie, and Jiningbairi, the primary trait was subtype I, while subtype II was the principal polydactyly phenotype in Houdans.

### Causative SNP found in Silkie is also completely associated with polydactyly in Beijing-You and Jiningbairi, but not in Houdan

**Fig 3:**
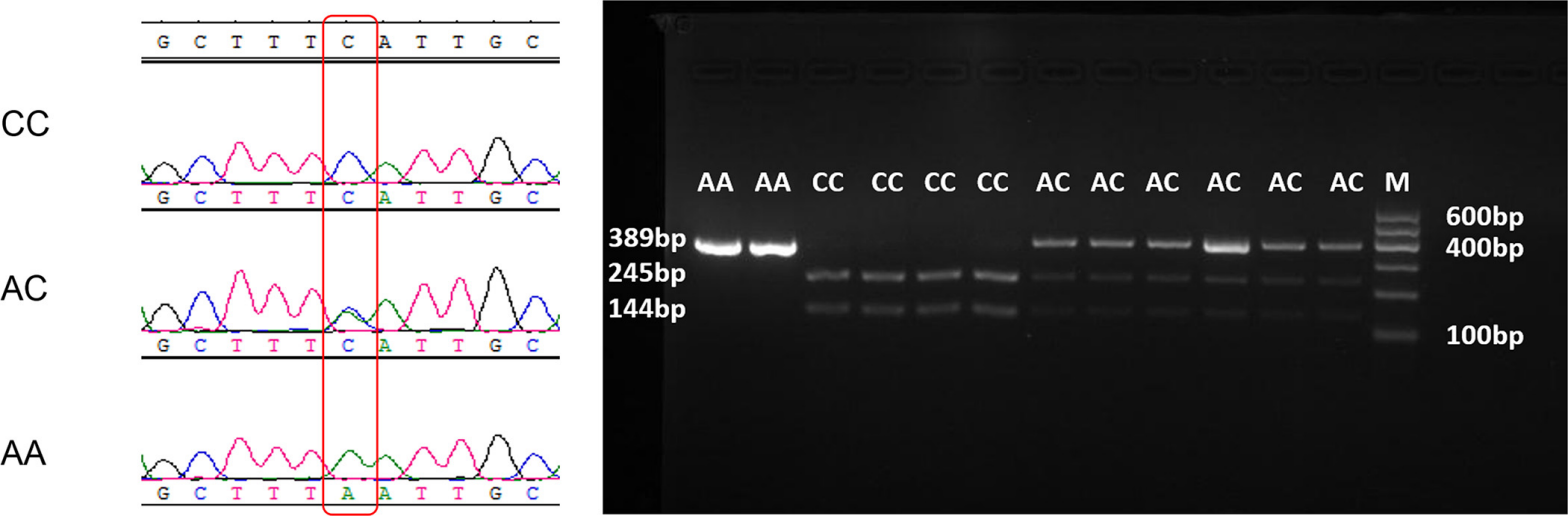
Genotypes of causative SNP loci detected by sequencing and diagnostic PCR. The genotypes shown on the left side are wild type (CC), mutation heterozygote (AC), and derived homozygote (AA), from top to bottom. The mutation genotypes were fully associated with polydactyly in Chinese chickens, regardless of whether the mutation was homozygotic or heterozygotic. PCR products digested by restriction enzyme BsrDI are presented on the right.

The reported causative SNP (ss161109890, C > A) in Silkie was also found in Beijing-You, Silkie, and Jiningbairi flocks, but not in Houdan flocks (Table 1 and Fig 3). We analyzed the association between this point mutation and polydactyly in Beijing-You, Silkie, and Jiningbairi. We found that when birds have the polydactyly phenotype (including polyphalange), the genotype on this locus is AA or AC (Fig 3), showing complete association with polydactylous birds (Table 1). The causative mutation was not found in any of the other 306 non-polydactyly chickens, which included the White Leghorn breed and 21 indigenous Chinese breeds, except for 1 heterozygous chicken from the Langya breed, which has been recorded to have a low frequency of polydactyly [37] (Table 1).

### Relationship of a ~0.39 Mb region to polydactyly in Houdan

**Fig 4:**
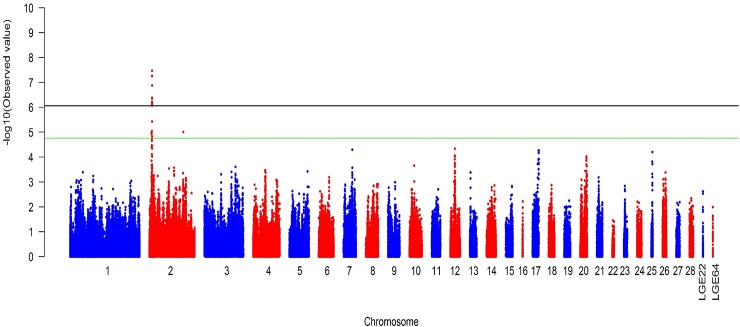
Genome-wide scan for polydactyly in Houdan chickens. Manhattan plot showing the association of all SNPs with the polydactyly trait of Houdan chickens. SNPs were plotted on the x-axis according to their position on each chromosome against their association with these traits on the y-axis (shown as -log10 P-value). The green line and black line indicate the genome-wide suggestive and significant association with P-values of 1.73 × 10^−5^ (1.00/57,657) and 8.67 × 10^−7^ (0.05/57,657), respectively.

To determine the molecular mechanism that contributes to polydactyly in European chickens, we performed GWAS on Houdan, as the representative of European chickens by using the 600 K chicken SNP array. We found that 24 SNPs (Table 2) located at 8.16–8.55 Mb on GGA2 in the current assembly of the chicken genome (galGal4) were significantly associated with polydactyly in Houdan, with the causative mutation in local Chinese breeds also being located in this region (Fig 4). In addition, 16 SNPs that were also clustered on GGA2 may be significantly associated with polydactyly (S1 Table). The 24 SNPs defined a 386.94 kb region (2:8165823–8552766), while the six involved genes were Sonic hedgehog (*SHH*), ENSGALG00000026913, RING finger protein 32 (*RNF32*), Limb region 1 protein homolog (*LMBR1*), Nucleolar protein with MIF4G domain 1 (*NOM1*), and Motor neuron and pancreas homeobox 1 (*MNX1*), also known as Homeobox Hb9 (*HLXB9*) (Table 2).

**Table 2:** Single nucleotide polymorphisms (SNPs) showing significant association with polydactyly in Houdans.

SNP	Chromosome	Position	Minor allele	Major allele	MAF	P value	Consequence	Nearest gene	Amino Acids	Codons
rs14135877	2	8544177	A	G	0.122	3.35E-08	Upstream gene variant	*MNX1*, U 2669 bp	-	-
rs314980016	2	8215183	A	G	0.116	5.51E-08	Intergenic variant	*ENSGALG00000026913*/*RNF32*	-	-
rs314255958	2	8459984	T	G	0.116	5.51E-08	Intron variant	*LMBR1*, intron 5/16	-	-
rs313395545	2	8471163	A	G	0.116	5.51E-08	Intron variant	*LMBR1*, intron 5/16	-	-
rs316170368	2	8538341	T	G	0.113	1.33E-07	Downstream gene variant	*MNX1*, D 527 bp	-	-
rs313083919	2	8544022	G	A	0.128	4.12E-07	Upstream gene variant	*MNX1*, U 2514 bp	-	-
rs313869663	2	8551817	T	C	0.128	4.12E-07	Intergenic variant	*MNX1*/UBE3C	-	-
rs313897469	2	8552766	A	G	0.141	4.46E-07	Intergenic variant	*MNX1*/UBE3C	-	-
rs316997633	2	8165823	C	T	0.134	6.09E-07	Intron variant	*ENSGALG00000026913*, 1/1 intron	-	-
rs316014656	2	8178077	G	A	0.134	6.09E-07	Intron variant	*ENSGALG00000026913*, 1/1 intron	-	-
rs14135527	2	8184950	C	T	0.134	6.09E-07	Downstream gene variant	*ENSGALG00000026913*, 1/1 intron	-	-
rs13536687	2	8350250	C	T	0.134	6.09E-07	Intergenic variant	*ENSGALG00000026913*/*RNF32*	-	-
rs15883157	2	8354321	G	C	0.134	6.09E-07	Intergenic variant	*ENSGALG00000026913*/*RNF32*	-	-
rs313549612	2	8383664	G	C	0.134	6.09E-07	Intergenic variant	*ENSGALG00000026913*/*RNF32*	-	-
rs316318736	2	8393250	G	A	0.134	6.09E-07	Intergenic variant	*ENSGALG00000026913*/*RNF32*	-	
rs318006969	2	8411941	C	T	0.134	6.09E-07	Upstream gene variant	*RNF32*, U 2505 bp	-	-
rs314624794	2	8426661	A	G	0.134	6.09E-07	Missense variant	*RNF32*, 7/8 exon	K/E	Aag/Gag
rs312318076	2	8427333	T	C	0.134	6.09E-07	Synonymous variant	*RNF32*, 8/8 exon	Y	taT/taC
rs13536758	2	8481851	G	A	0.134	6.09E-07	Intron variant	*LMBR1*, intron 2/16	-	-
rs315933374	2	8407928	T	C	0.122	6.96E-07	Intergenic variant	*ENSGALG00000026913*/*RNF32*	-	-
rs14135799	2	8442074	C	T	0.122	6.96E-07	Intron variant	*LMBR1*, intron 13/16	-	-
rs15883517	2	8447165	T	C	0.122	6.96E-07	Intron variant	*LMBR1*, intron 9/16	-	-
rs15882649	2	8180068	C	T	0.135	7.48E-07	Intron variant	*ENSGALG00000026913*, 1/1 intron	-	-
rs314223644	2	8485974	C	T	0.135	8.41E-07	Intron variant	*LMBR1*, intron 1/16	-	-

### Parallel evolution of polydactyly traits in Chinese and European chickens

We demonstrated that polydactyly phenotypes in indigenous Chinese breeds and Houdans have different molecular mechanisms. The causative SNP in Silkie had 100% association with polydactyly in all Chinese indigenous chicken breeds, but not in Houdans or other European breeds, such as Salmon Faverolle and Silver Gray Dorking [15]. This result indicates that the polydactyly phenotypes in Chinese and European chickens were caused by different mutation events, providing another example of parallel evolution in vertebrates.

## Discussion

The causative SNP in Silkie completely associated with polydactylous Beijing-You and Jiningbairi, indicating that the SNP is also causative for polydactyly in Beijing-You and Jiningbairi. For Silkie, our result supports the previous study on other Silkie flocks [26]. For Beijing-You, our results are more substantial than previous studies, in which the polydactyly loci were mapped in special regions and several candidate association genes were identified [27–31]. Our results also identified the causative mutation of polydactylous Silkie was not exist in other nonpolydactyly chickens, suggesting that SNP of polydactyly in Silkie has fully penetrated all other indigenous Chinese chicken breeds.

In this study, the causative SNP in polydactylous Silkie was not detected in our Houdan flocks. Previously, the SNP was confirmed as the causative mutation of polydactyly in Silkie [26]. Furthermore, another study on five polydactylous chicken breeds (Silkie, Salmon Faverolle, Mottled Houdan, Silver Gray Dorking, and White Sultan) found that the same SNP (ss161109890) is highly associated with polydactyly in Silkie and Sultan breeds, but could not be detected in the other three polydactylous breeds [15]. On investigating the origin of these five breeds, we found that the Silkie originates from China, whereas the White Sultan originates from Turkey (Asia) [38]. In addition, Salmon Faverolle and Mottled Houdan originate from France (Europe), while the Silver Gray Dorking originates from Italy (Europe) [38]. Thus, the principal subtype of the polydactyly phenotype differs between European [15] and Chinese breeds, and even between European and Asian breeds. We hypothesize that the molecular mechanism of polydactyly in Chinese and Asian chickens may differ to that of European chickens.

To determine the molecular mechanism that contributes to polydactyly in European chickens, we performed GWAS on Houdan, as the representative European chicken breed. The GWAS results identified 24 SNPs in a ~0.39 Mb region on GGA2, and identified 6 candidate genes: *SHH*, *ENSGALG00000026913*, *RNF32*, *LMBR1*, *NOM1*, and *MNX1/HLXB9*. The *RNF32* gene is one of the *cis*-regulators of *SHH* in the mouse [39]. This regulatory element directs the initiation and spatial expression of *SHH* [40]. *SHH* is one of the proteins of the mammalian signaling pathway family hedgehog [41]. *SHH* is the best studied ligand of the hedgehog signaling pathway, because of its key role in regulating vertebrate organogenesis [42], such as the growth of digits on limbs [43] and the organization of the brain [44]. We speculate that the SNPs close to or in *RNF32* may be critical for regulating the temporal and spatial expression of *SHH*, which contributes to polydactyly in Houdans.

The *LMBR1* gene is one of the *cis*-regulators of *SHH*, and is critical for *SHH* expression [11, 16]. The mutations in *LMBR1* may directly cause polydactyly in vertebrates. Thus, a point mutation in the ZRS may directly regulate the expression of *SHH*, and, consequently, the occurrence of polydactyly or other types of limb malformation [22, 24, 25]. For instance, different SNPs in intron 5 of the *LMBR1* gene may lead to triphalangeal thumb-polysyndactyly syndrome in humans [45] and polydactyly in Silkie chickens [26]. Deletions in exon 4, and in portions of intron 3 and 4 of chicken *LMBR1*, may cause the oligozeugodactyly mutant phenotype [13], which is similar to that of humans affected with acheiropodia [46].

Compared with genes detected in previous studies on chicken polydactyly, the *MNX1/HLXB9* gene is a novel candidate gene associated with chicken polydactyly [29, 30, 47]. In humans, the *MNX1/HLXB9* gene is hypothesized to be a candidate gene for polydactyly [48, 49]. Mutations in the *MNX1/HLXB9* gene may cause neonatal diabetes and Currarino syndrome [50, 51]. Currarino syndrome is a malformation with an autosomal dominant genetic trait similar to polydactyly [52–54]. However, the function of *MNX1/HLXB9* in the chicken has not been documented yet. Thus, this gene may also affect skeletal development, including the incidence of polydactyly in chickens.

In this study, the causative SNP in Silkie was validated be the causative mutation of all Chinese indigenous chicken breeds, but not of Houdan or the other European chicken breeds, such as Salmon Faverolle and Silver Gray Dorking [15]. This result indicates that the polydactyly phenotypes in Chinese and European chickens are caused by different mutation events, providing another example of parallel evolution in vertebrates. Parallel evolution is ubiquitous in living organisms [55]. For instance, the blue eggshell of Chinese, South American, and European chickens is caused by an EAV-HP insertion in the 5ʹ flanking region of *SLCO1B3*; however, the retrovirus integration site of American and European chickens differs from that of Chinese chickens [56, 57]. As another example, the black coat color of Asian and European domestic pigs is caused by independent missense mutations in melanocortin 1 receptor (*MC1R*), which occurred on different haplotypes originating from Asian and European wild boar [58].

In conclusion, we confirm that the genetic basis of chicken polydactyly traits differs between Chinese and European breeds, providing a clear example of parallel evolution. The results of this study are expected to facilitate further studies on limb malformation in chickens, which could be extrapolated to other vertebrates.

## Supporting Information

S1 Table Summary of identified SNP association for polydactyly in Houdan chickens. (XLSX)
